# Development and Temporal Validation of Explainable Machine Learning Models for Predicting Vitamin B12 Deficiency Using Routine Laboratory Analytes

**DOI:** 10.3390/diagnostics16040563

**Published:** 2026-02-13

**Authors:** Ferhat Demirci, Oktay Yıldırım, Aylin Demirci, Pınar Akan

**Affiliations:** 1 Department of Medical Biochemistry, İzmir Tepecik Training and Research Hospital, University of Health Sciences Türkiye, Gaziler Street 468, Yenişehir, Konak, 35120 İzmir, Türkiye; 2 Department of Neurosciences, Institute of Health Sciences, Dokuz Eylül University, 35340 İzmir, Türkiye; 3 Information Technology Department, Dokuz Eylül University Rectorate, 35340 İzmir, Türkiye; 4 Department of Family Medicine, İzmir Tepecik Training and Research Hospital, University of Health Sciences Türkiye, Gaziler Street 468, Yenişehir, Konak, 35120 İzmir, Türkiye; 5 Department of Medical Biochemistry, Faculty of Medicine, Dokuz Eylül University, 35340 İzmir, Türkiye

**Keywords:** vitamin B12 deficiency, machine learning, explainable artificial intelligence, CatBoost, laboratory diagnostics, clinical decision support

## Abstract

**Background/Objectives:** Vitamin B12 deficiency is a prevalent yet frequently underdiagnosed condition, largely due to the limited diagnostic accuracy of serum total B12 and the restricted availability of confirmatory biomarkers such as holotranscobalamin and methylmalonic acid. This study aimed to develop and validate explainable machine learning (ML) models capable of predicting vitamin B12 deficiency using only routinely available laboratory examinations, thereby supporting early detection within standard diagnostic workflows. **Methods:** This retrospective study included 51,630 adult patients who underwent concurrent vitamin B12 testing and routine laboratory evaluation between 2015 and 2025. An independent temporal validation cohort of 34,744 patients was used to assess generalizability. Eight supervised ML algorithms were developed within a four-stage experimental framework incorporating default modeling, probability-threshold optimization, hyperparameter tuning, and feature engineering. Model performance was evaluated using AUC-ROC, AUC-PR, sensitivity, specificity, F1 score, accuracy, Matthews correlation coefficient, and likelihood ratios. Model explainability and clinical utility were assessed using SHAP, LIME, and decision curve analysis. **Results:** Among all algorithms, CatBoost demonstrated the most balanced and clinically relevant performance. In the threshold-optimized configuration, the model achieved a sensitivity of 0.92, specificity of 0.67, F1 score of 0.82, AUC-ROC of 0.88, and AUC-PR of 0.86 in the test set. Temporal validation confirmed robust generalizability, with improved discrimination (AUC-ROC 0.90; AUC-PR 0.91) and stable calibration. Explainability analyses identified hematologic indices (MCV, HGB, HCT, RDW), iron-related markers, inflammatory measurands, and age as the most influential contributors, consistent with known pathophysiology. **Conclusions:** This study presents a large-scale, explainable, and temporally validated ML framework for predicting vitamin B12 deficiency using routine laboratory data alone. The model demonstrates strong diagnostic performance, biological plausibility, and potential for seamless integration into laboratory and clinical decision-support systems, enabling cost-effective and early identification of patients at risk.

## 1. Introduction

Vitamin B12 (cobalamin) is an essential micronutrient required for DNA synthesis, myelin maintenance, and one-carbon metabolism. Deficiency can result in macrocytic anemia, neurocognitive impairment, and increased cardiovascular risk mediated through hyperhomocysteinemia [1]. Although serum total B12 concentration is the most frequently used diagnostic test, it often fails to reflect intracellular cobalamin status, leading to “functional B12 deficiency” even when serum levels fall within the low-normal range [1,2]. More specific biomarkers—such as holotranscobalamin (holo-TC), methylmalonic acid (MMA), and homocysteine (Hcy)—provide improved diagnostic accuracy, yet their higher cost, limited availability, and lack of integration into routine laboratory workflows restrict widespread use [3,4]. Consequently, clinically significant deficiency may remain undetected until hematological or neurological manifestations become evident [2].

In recent years, the growing availability of large-scale laboratory datasets and advances in machine learning (ML) have opened new possibilities for leveraging routinely collected biochemical and hematological measurands to predict micronutrient deficiencies. Tamune et al. demonstrated that ML models using standard blood tests can efficiently predict vitamin B group deficiencies, including B12, in patients with acute psychiatric episodes [5]. Beyond B12, several studies have applied ML to other micronutrients and lipid biomarkers: Sharifmousavi and Borhani developed an SVM-based model that incorporated selenium, vitamin B12, and vitamin D3 levels for the diagnosis of multiple sclerosis [6], while Sancar and Tabrizi showed that ML approaches can accurately classify vitamin D status using routine laboratory variables [7]. Similarly, ML-based models have been proposed for predicting low-density lipoprotein cholesterol (LDL-C) from standard biochemical profiles, achieving good agreement with directly measured and calculated LDL-C values [8]. Collectively, these findings highlight the feasibility of using routinely available laboratory data to build predictive models for diverse biochemical targets.

Another important development has been the increasing emphasis on explainable artificial intelligence (XAI) in clinical prediction models. Hu et al. reported an explainable ML model for predicting spontaneous bacterial peritonitis in cirrhotic patients with ascites, using SHAP-based explanations to identify the most influential laboratory and clinical variables. In a broader perspective, a recent systematic review by Alkhanbouli et al. underscored the role of explainable AI techniques in disease prediction and clinical decision support, emphasizing transparency, trust, and model interpretability as essential requirements for implementation in healthcare settings [9]. For high-stakes decisions such as the detection of vitamin B12 deficiency—where underdiagnosis may lead to irreversible neurological damage—models that are both accurate and interpretable are particularly valuable.

Given the high prevalence of vitamin B12 deficiency, its often subtle or delayed clinical presentation, and the limited accessibility of confirmatory biomarkers, there is a clear need for robust, low-cost, and scalable predictive models based entirely on routine laboratory measurands. Such models could be integrated into hospital information systems as automated decision-support tools, providing real-time alerts for individuals at high risk and reducing unnecessary second-line testing.

The present study aims to address these gaps by developing and validating several machine learning algorithms—including artificial neural networks, boosting-based ensemble models, support vector machines, and logistic regression—to predict laboratory-defined low vitamin B12 status using a large, multiyear dataset of routinely collected biochemical and hematological measurands. Model performance was evaluated using ROC-AUC, PR-AUC, F1-score, accuracy, and the Matthews correlation coefficient. To ensure clinical interpretability, SHAP and LIME techniques were employed to identify the variables contributing most substantially to model predictions. Furthermore, an independent temporal validation cohort was used to assess the generalizability and robustness of the final model under real-world conditions. Overall, this study aims to establish a reliable, scalable, and explainable machine learning framework capable of supporting early identification of individuals at risk for vitamin B12 deficiency within routine clinical practice.

## 2. Materials and Methods

### 2.1. Study Population/Subjects

This retrospective study included adult patients who underwent serum vitamin B12 testing together with routine hematological and biochemical examinations at Health Sciences University İzmir Tepecik Training and Research Hospital between 1 January 2015 and 1 October 2025. Prior to study initiation, approval was obtained from the hospital’s Non-Interventional Research Ethics Committee (initial ethics approval: 13 July 2023—2023/06-38; additional approval: 30 October 2025—293077648). Laboratory data were extracted from the hospital information system (HIS) and were based on instrument outputs verified and authorized by a board-certified medical biochemistry specialist. Only the first eligible laboratory encounter for each patient was included in the study, and all records were anonymized during data extraction. Only outpatient laboratory encounters were included in this study. Inpatient laboratory data were intentionally excluded in order to minimize potential confounding effects related to acute illness severity, ongoing medical treatments, intravenous fluid administration, and rapid physiological fluctuations, which may substantially influence hematological and biochemical parameters independent of vitamin B12 status. By restricting the analysis to the first eligible outpatient laboratory encounter for each patient, we aimed to ensure greater biological stability of the measured variables and to improve the clinical interpretability and real-world applicability of the developed machine-learning models.

### 2.2. Inclusion Criteria

- Age ≥ 18 years
- Availability of serum vitamin B12 measurement performed concurrently with all predefined hematological and biochemical tests
- First eligible outpatient laboratory encounter within the study period
- Complete numerical laboratory results available for all required parameters
- Specimen processed within the institution’s standardized pre-examination workflow and quality limits

### 2.3. Exclusion Criteria

- Age < 18 years
- Missing, corrupted, or non-numerical laboratory results
- Multiple encounters for the same patient, in which case only the first eligible record was retained
- Specimens exhibiting pre-examination delays exceeding institutional workflow limits
- Pregnancy
- Active oncologic disease or hematologic malignancy
- Forensic/medico-legal cases
- Emergent conditions associated with spontaneous acute bleeding or requiring immediate emergency evaluation, including but not limited to:
  - Acute coronary syndrome
  - Pulmonary embolism
  - Aortic dissection/rupture
  - Subacute arachnoid hemorrhage
  - Transient ischemic attack
  - Massive gastrointestinal hemorrhage

Pregnancy, active oncologic disease, hematologic malignancy, and acute emergent conditions were excluded because these states are known to induce substantial physiological and treatment-related alterations in hematological indices and biochemical parameters, independent of vitamin B12 status. Such alterations may introduce confounding variability and obscure the underlying relationship between routine laboratory measurands and vitamin B12 deficiency. Excluding these conditions was therefore intended to improve the biological validity and interpretability of the machine-learning models.

This cohort represents a clinically enriched population in which serum vitamin B12 testing was performed based on clinical suspicion rather than population-wide screening. Accordingly, the model is intended for risk stratification and decision support within routine diagnostic workflows, and not for indiscriminate population screening. Accordingly, the observed prevalence of vitamin B12 deficiency in this cohort should not be interpreted as population prevalence but rather as reflecting a clinically selected diagnostic setting.

Venous whole-blood samples were collected into K_2_-EDTA tubes, and serum samples were obtained in additive-free plain tube, in accordance with routine clinical protocols. In line with the National Health Quality Standards (Version 5.1), all specimens were delivered to the laboratory within 30 min, and biochemical measurements were completed within two hours of sample collection.

Hematological measurands, including hemoglobin (HGB), red blood cell count (RBC), hematocrit (HCT), mean corpuscular volume (MCV), red cell distribution width (RDW), mean corpuscular hemoglobin (MCH), mean corpuscular hemoglobin concentration (MCHC), white blood cell count (WBC), neutrophil count (NEU), lymphocyte count (LYM), monocyte count (MONO), basophil count (BASO), platelet count (PLT), mean platelet volume (MPV), and platelet distribution width (PDW), were measured using LH-780 (Beckman Coulter Inc., Mannheim, Germany) and XN-1000 (Sysmex Corporation, Kobe, Japan) hematology analyzers. Biochemical assays, including glucose, creatinine, alanine aminotransferase (ALT), aspartate aminotransferase (AST), alkaline phosphatase (ALP), gamma-glutamyl transferase (GGT), lactate dehydrogenase (LDH), serum iron, C-reactive protein (CRP), albumin, total protein, and total bilirubin, were performed on an AU5800 analyzer (Beckman Coulter Inc., Mannheim, Germany). Chemiluminescence-based immunoassays, including vitamin B12, folate, ferritin, free thyroxine (fT4), and thyroid-stimulating hormone (TSH), were conducted on the DxI800 analyzer (Beckman Coulter Inc., Mannheim, Germany).

All reagents, calibrators, and internal quality-control materials were certified and approved by the manufacturer. Standard institutional internal and external quality-control procedures were maintained throughout the examination process. All laboratory results were reviewed and analytically validated by a medical biochemistry specialist before being transferred to the hospital information system.

### 2.4. Study Design

To ensure data confidentiality, all patient identifiers were irreversibly removed prior to analysis. A comprehensive dataset containing age, gender, and all hematological and biochemical measurands was generated through the hospital information system (HIS). A total of 87,301 laboratory records from the study period were reviewed and exported to Microsoft Excel 2021 (USA). After applying the predefined exclusion criteria, 51,630 patients were deemed eligible and included in the study, while all other records were excluded.

To address class imbalance in the machine-learning analyses and enable the model to effectively learn the minority class, a random undersampling strategy was applied to construct the development dataset. This procedure was performed in Python, whereby all observations belonging to the minority class were retained, and an equal number of records were randomly selected from the majority class. As a result of this class-balancing step, 16,354 records from the majority class were randomly discarded, yielding a final development cohort of 35,276 patients. Class balancing was applied exclusively during model development to improve learning of the minority class. All reported performance metrics were interpreted with careful consideration of the underlying class distribution, and final model evaluation was performed on an independent temporal validation cohort without any modification of the trained model. This intentional 1:1 class ratio was implemented solely to facilitate algorithmic learning and to reduce the risk of overfitting and majority-class dominance; it does not represent the underlying clinical prevalence of vitamin B12 deficiency in the source population.

The development dataset (1 January 2015–30 May 2023) was subsequently split into 80% training and 20% test sets using stratified random partitioning, in order to preserve class distribution and reduce the risk of overfitting. Using identical preprocessing steps and the same random under sampling strategy, an independent temporal validation set consisting of 34,744 patient records (collected between 1 June 2023 and 1 October 2025) was used for external validation. This approach enabled evaluation of the model’s temporal generalizability on data derived from a later clinical period. The temporal validation cohort was evaluated without any additional class balancing or recalibration, allowing assessment of model performance under a naturally evolving clinical data distribution.

**Figure 1:**
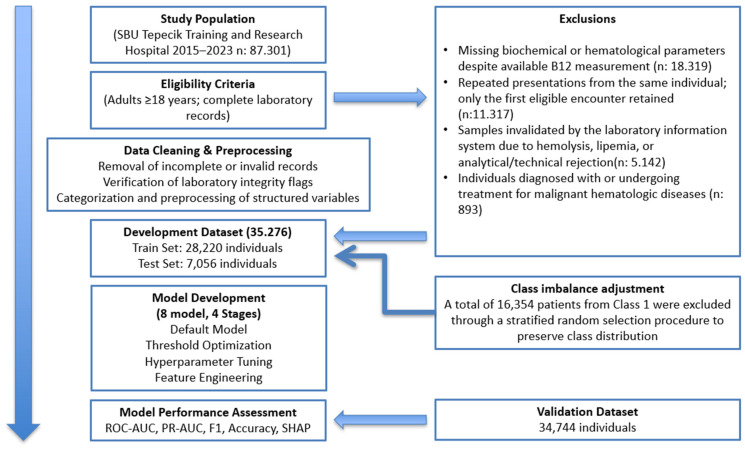
Study Flow Diagram for Cohort Selection and Data Processing.

All data preprocessing, model development, class balancing, training–test partitioning, and reporting procedures were conducted in accordance with the TRIPOD guidelines. A flow diagram summarizing patient selection, exclusion criteria, and the final analytic cohorts is presented in Figure 1.

### 2.5. Data Preprocessing and Training of Machine-Learning Algorithms

All preprocessing steps—including removal of duplicate records, unit standardization, exclusion of entries with missing values, categorical encoding based on reference intervals, and feature engineering—were predefined and applied to the dataset *prior to splitting* into training, test, and temporal validation sets. This ensured a consistent and reproducible preprocessing pipeline across all data subsets and prevented discrepancies arising from heterogeneous data preparation.

Raw laboratory data extracted from the HIS were preprocessed through removal of duplicate patient records, standardization of unit inconsistencies for selected biochemical measurands (e.g., albumin, total protein), and exclusion of entries containing missing parameters. Given that extreme values may represent true biological measurements within clinical practice and can directly influence model performance, outliers were not removed. This approach was adopted to preserve real-world clinical variability and avoid discarding clinically meaningful extremes. To mitigate potential sensitivity of certain algorithms to extreme values, the only continuous variable (age) was standardized prior to model training. This strategy is consistent with prior clinical machine-learning studies emphasizing the importance of retaining biologically plausible extreme values to enhance generalizability [10].

Age was retained as a continuous variable, whereas all biochemical and hematological analytes were transformed into categorical variables based on clinically defined threshold values. Evidence-based clinical decision limits were used when available; otherwise, manufacturer-provided reference intervals were applied. Each analyte was encoded as 0 (below reference interval), 1 (within reference interval), or 2 (above reference intyerval). For parameters without a biologically meaningful lower bound, a binary scheme was adopted. Gender was coded as 1 for males and 2 for females.

Serum vitamin B12 concentrations were initially classified into three categories according to established clinical guidelines: deficiency (<180 pg/mL), possible vitamin B12 deficiency (180–350 pg/mL), and normal status (>350 pg/mL). These thresholds reflect clinically meaningful decision limits and are consistent with international recommendations (e.g., NICE guidelines [11]).

For the primary machine-learning analyses, the initial three-category structure of vitamin B12 was subsequently converted into >350 a binary variable (0 = deficient/insufficient (<350 pg/mL); 1 = within reference interval (>350 pg/mL). This transformation was performed to reduce class imbalance and to reflect real-world clinical practice, in which patients with borderline vitamin B12 levels are often managed similarly to those with overt deficiency. Classification thresholds for all measurands are provided in Supplementary Material S1, Table S1.

Eight supervised machine-learning algorithms—logistic regression, random forest, decision tree, support vector machines, k-nearest neighbors, XGBoost, CatBoost, and artificial neural networks—were developed within a four-stage experimental framework. These algorithms were selected to represent a diverse set of supervised learning paradigms commonly used in clinical prediction modeling. Logistic regression was included as a transparent linear baseline model, whereas decision tree and random forest algorithms were used to capture non-linear relationships and feature interactions. Support vector machines and k-nearest neighbors represent kernel-based and distance-based learning strategies, respectively, but may be sensitive to feature scaling and data dimensionality. Gradient boosting methods (XGBoost and CatBoost) were selected due to their strong performance on structured tabular data, with CatBoost offering additional robustness through built-in regularization. Artificial neural networks were included to model complex non-linear patterns despite their lower interpretability. Other model families were not considered to avoid unnecessary methodological complexity and to maintain a focused, interpretable comparison aligned with current best practices in clinical machine-learning research [12]. In experiment 1, all algorithms were trained using default hyperparameters. In experiment 2, probability-threshold optimization was performed using the Youden J index and F1-maximization criterion. The Youden J index was used to identify the optimal classification threshold by maximizing the sum of sensitivity and specificity, which is a widely accepted approach in diagnostic accuracy studies. In parallel, F1-score maximization was applied to balance precision and recall, particularly in the presence of class imbalance. These complementary strategies ensure robust threshold selection from both diagnostic and machine-learning perspectives [13,14]. Experiment 3 involved extensive hyperparameter tuning conducted exclusively within the training set after the initial train–test split to prevent information leakage. Cross-validation was applied only to internal folds of the training data, while the test set and the independent temporal validation cohort were reserved solely for performance evaluation. Hyperparameter optimization was performed sequentially: GridSearchCV was used for exhaustive tuning of a limited set of key hyperparameters, followed by RandomizedSearchCV for stochastic exploration of broader parameter spaces. Subsequently, Optuna-based Bayesian optimization was employed to efficiently refine hyperparameters in high-dimensional search spaces by iteratively updating probabilistic performance estimates. These complementary approaches are widely used in machine-learning research to improve model generalization and reduce overfitting [15]. All cross-validation procedures were conducted exclusively on the training set to prevent information leakage. In experiment 4, a large-scale feature-engineering pipeline generated more than 250 derived variables, including variance-based metrics, ratio-based biomarkers, composite indices, and biologically meaningful interaction terms (Supplementary Material S2). No explicit feature selection or dimensionality reduction techniques (such as principal component analysis) were applied after feature generation. This decision was motivated by the use of algorithms with built-in regularization and feature selection properties, particularly tree-based ensemble and gradient boosting methods. Potential overfitting was further controlled through strict separation of training, test, and independent temporal validation datasets, as well as through cross-validation and hyperparameter optimization performed exclusively on the training set. The generalizability of the expanded feature space was ultimately assessed using an external temporal validation cohort.

All preprocessing and model-building steps were implemented in Python 3.11 (The Netherlands) using Kaggle Notebook and Google Colab environments. Age was the only variable retained in continuous form and was therefore the only feature subjected to standardization using the StandardScaler. Continuous variables were standardized using the StandardScaler function, and the final dataset was randomly split into training (80%) and test (20%) subsets using a stratified approach to preserve the distribution of vitamin B12 classes.

After completion of all stages of the experimental framework, the methodological strategy yielding the highest overall performance was selected and applied—without modification—to an independent temporal validation cohort of 34,744 patients from a later time period, in order to assess real-world, time-dependent generalizability. Model interpretability and clinical utility were comprehensively evaluated using SHAP (feature contributions), LIME (local explainability), and Decision Curve Analysis (net clinical benefit).

### 2.6. Performance Evaluation

Descriptive analyses were performed for both the development and validation cohorts. Continuous variables were summarized as mean ± standard deviation, and between-group differences were assessed using *p*-values and Cohen’s d. Additional stratified analyses by age and sex were conducted separately for the development and validation datasets. Subsequently, the performance of the eight algorithms included in the four-stage experimental framework was evaluated using sensitivity, specificity, positive and negative predictive values, accuracy, F1 score, Matthews correlation coefficient, area under the ROC curve (AUC-ROC), and area under the precision–recall curve (AUC-PR), the latter being particularly informative for imbalanced outcomes. For each model, confusion-matrix components (TP, FP, TN, FN) were computed, and ROC/PR curves were generated across a range of probability thresholds. Because positive and negative predictive values, likelihood ratios, and decision curve analyses are sensitive to outcome prevalence, these metrics were interpreted in the context of the study’s clinically enriched cohort and the applied class-balancing strategy during model development.

After completion of all experimental stages, the methodological strategy demonstrating the highest overall performance on the test set was selected and applied unchanged to the validation cohort. Clinical interpretability was assessed using SHAP beeswarm plots, LIME-based local explanations, and Decision Curve Analysis, providing complementary insights into feature contributions and net clinical utility.

Throughout the analyses, the categorical vitamin B12 variable was used exclusively as the ground-truth outcome label (0 = deficient/insufficient, 1 = within reference interval). Serum vitamin B12 was not included among the model input features at any stage of model development, training, or evaluation. These two representations were explicitly distinguished across all analytical steps to avoid interpretive ambiguity.

### 2.7. Statistical Comparison of Experimental Models

Since all machine-learning algorithms were evaluated on the same test set, statistical comparisons accounted for the dependence arising from correlated prediction structures. DeLong’s test was used to compare AUC-ROC values derived from correlated ROC curves. Differences in continuous performance metrics such as F1 score and accuracy were assessed using paired *t*-tests.

To examine false-positive/false-negative imbalance and asymmetry in misclassification patterns across models, the McNemar test was applied. In addition, Net Reclassification Improvement (NRI) and Integrated Discrimination Improvement (IDI) were calculated to quantify the relative gains in reclassification performance between models. These metrics enabled a more comprehensive evaluation of the methodological advantages achieved through model optimization, particularly regarding improvements in accuracy and discrimination.

Together, these complementary procedures allowed for a rigorous, multidimensional, and model-agnostic comparison of all experimental configurations.

### 2.8. Statistical Analysis

Continuous variables were summarized as mean ± standard deviation, whereas categorical variables were presented as counts and percentages. Given the large sample size, parametric methods were preferred. Group comparisons for continuous variables were performed using one-way ANOVA, and Welch’s ANOVA was applied when the assumption of homogeneity of variances was violated. When appropriate, Tukey’s HSD or Games–Howell tests were used for post hoc multiple comparisons. Categorical variables were compared using the Pearson chi-square test.

The diagnostic performance of the machine-learning models was assessed using sensitivity, specificity, positive and negative predictive values, accuracy, F1 score, AUC-ROC, AUC-PR, positive and negative likelihood ratios (LR^+^/LR^−^), and the Matthews correlation coefficient. All statistical analyses were conducted in Python 3.11 using the *pandas*, *scipy*, *scikit-learn*, and *statsmodels* libraries. A two-sided *p*-value < 0.05 was considered statistically significant.

## 3. Results

Statistically significant differences were observed across numerous hematological and biochemical measurands between the development (n = 35,256) and independent validation (n = 34,744) cohorts (with *p* < 0.05 for most comparisons). However, Cohen’s d analyses indicated that the magnitude of these differences was predominantly very small from a biological perspective. Age, hemoglobin, hematocrit, erythrocyte count, and MCV values were lower in the validation cohort, demonstrating small to moderate effect sizes (d = 0.18–0.33). In contrast, parameters reflecting inflammatory or hematologic variability—such as RDW, CRP, LDH, and PLT—were higher, corresponding to small effect sizes (d ≈ −0.16 to −0.20). Among liver enzymes, only ALP exhibited a moderate effect size at the cohort level (d = −0.546). Although serum vitamin B12 concentrations were higher in the validation period, the biological magnitude of this difference remained very small (d = −0.143). Means, standard deviations, *p*-values, and Cohen’s d effect sizes for all variables are presented in Table 1. Additionally, age- and sex-stratified distributions and comparative statistics for both cohorts are provided in Supplementary Material S1, Tables S2 and S3.

Following binary transformation of the original three-category vitamin B12 classification, the development dataset was balanced using random undersampling, resulting in an equal distribution of vitamin B12–deficient/insufficient and reference-interval groups (50% each). All machine-learning models were trained and evaluated using this balanced binary outcome.

Across the eight machine-learning algorithms evaluated within the four-stage experimental framework, CatBoost demonstrated the most balanced sensitivity–specificity profile and the highest overall classification performance in all experiments. Notably, the threshold-optimized configuration (Experiment 2), despite yielding performance metrics numerically similar to the other strategies, provided a clinically decisive advantage. This approach improved model accuracy not only from a statistical perspective but also in terms of clinical safety, achieving the lowest number of false negatives and thereby minimizing the risk of missing true vitamin B12 deficiency cases. Given the association of vitamin B12 deficiency with neurological sequelae and irreversible damage, this marked reduction in false negatives represents the key outcome defining the model’s clinical applicability rather than merely its methodological strength.

For this reason, the primary findings highlighted in the manuscript are derived from the F1-maximization–based threshold-optimized configuration, which is summarized in Table 2. Performance distributions for all experimental strategies are provided in Supplementary Material S1, Tables S4–S6.

In the test set, the threshold-optimized CatBoost model achieved sensitivity 0.92, specificity 0.67, F1 score 0.82, PPV 0.73, and NPV 0.88, yielding the most balanced performance among all models; AUC-ROC was 0.88 and AUC-PR was 0.86. XGBoost and the neural network produced comparable AUC values but demonstrated lower accuracy, specificity, MCC, and PPV. Logistic regression and random forest failed to maintain overall classification balance, whereas SVM, KNN, and decision tree models showed markedly reduced specificity and MCC.

Performance metrics for the four CatBoost configurations are summarized in Table 3. Statistical comparisons revealed no significant differences across configurations for AUC-ROC (DeLong test) or for F1 and accuracy (paired *t*-tests) (all *p* > 0.05). In contrast, the McNemar test demonstrated a clear advantage for the threshold-optimized model, particularly through its favorable asymmetry in the distribution of false negatives versus false positives. This model substantially reduced false-negative decisions—the clinically most critical error type—supporting its selection as the final model.

For the threshold-optimized CatBoost model, the calculated NRI = 0.359 and IDI = 0.147 further confirmed that the approach produced meaningful improvements in both correct reclassification and discrimination capacity. These findings align with the McNemar test results demonstrating improved false-negative/false-positive asymmetry, collectively indicating that the optimized model provides a substantially enhanced risk-classification framework. Accordingly, the model exhibits strong potential as a reliable and effective clinical decision-support tool for the early identification of vitamin B12 deficiency.

**Figure 2:**
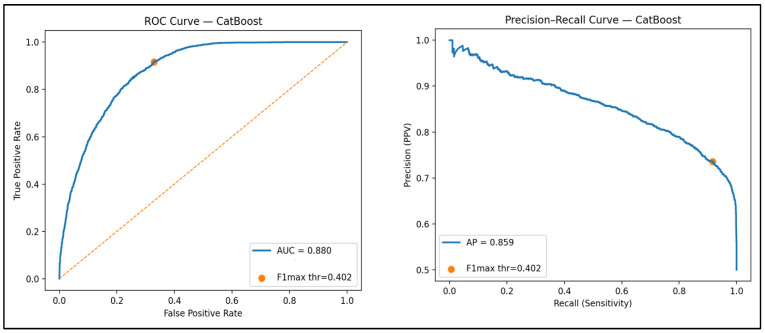
ROC and Precision–Recall curves of the threshold-optimized CatBoost model on the test set. ROC: Receiver Operating Characteristic; PR: Precision–Recall; AUC: area under the ROC curve; AP: average precision (area under the PR curve). The orange marker indicates the optimal probability threshold selected using the F1-maximization criterion (thr = 0.402). In this context, false-negative predictions are considered more clinically consequential than false positives, as false positives primarily lead to additional laboratory evaluation, whereas false negatives may delay recognition of a potentially reversible condition. In the ROC plot, this threshold corresponds to the operating point balancing sensitivity and specificity; in the PR curve, the same threshold reflects the precision–recall trade-off at the point of maximal F1 performance.

The performance of the selected CatBoost model remained robust in the independent temporal validation cohort, with sensitivity 0.85, specificity 0.77, accuracy 0.81, F1 score 0.82, and MCC 0.63. Discrimination improved compared with the internal test set, with AUC-ROC 0.90 and AUC-PR 0.91. The likelihood-ratio profile (PLR = 3.74; NLR = 0.19) also indicated strong clinical utility under real-world conditions. These results are presented in Table 4 and Figure 2.

**Figure 3:**
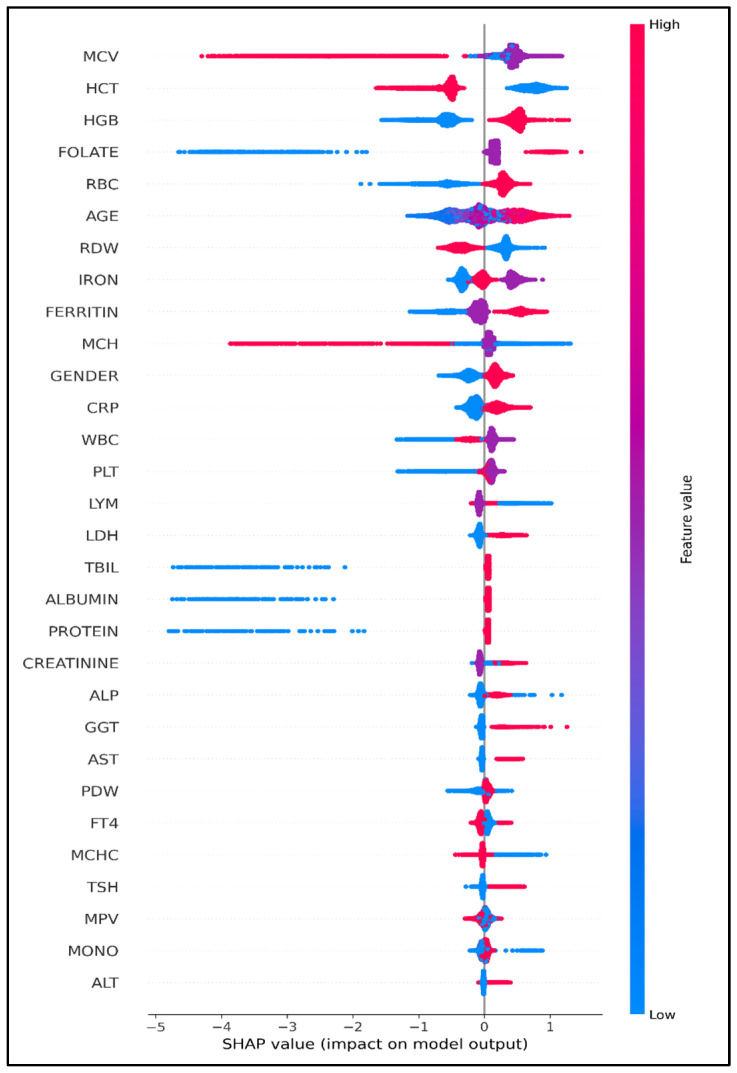
Global feature importance visualization using SHAP beeswarm for the final CatBoost classifier. SHAP: Shapley Additive Explanations. Each point represents a single observation, with color indicating the underlying feature value (red = high, blue = low). The position on the x-axis reflects the SHAP value, which quantifies the direction and magnitude of each feature’s contribution to the model output. Positive SHAP values shift the prediction toward vitamin B12 deficiency, whereas negative values shift the prediction toward the non-deficient class. The vertical spread demonstrates the variability of feature effects across individuals.

SHAP analyses demonstrated that hematologic and inflammatory measurands—such as MCV, HGB, HCT, RBC, RDW, age, iron and ferritin—were the strongest contributors to model predictions. The SHAP beeswarm plot (Figure 3) showed that physiologically abnormal measurements consistently shifted predictions toward deficiency. From a clinical perspective, commonly used rule-based indicators such as macrocytosis or anemia reflect late or incomplete manifestations of vitamin B12 deficiency and may fail to identify early or subclinical cases. By integrating multidimensional routine laboratory information, the proposed model provides incremental decision support beyond single-parameter heuristics and complements conventional clinical assessment.

**Figure 4:**
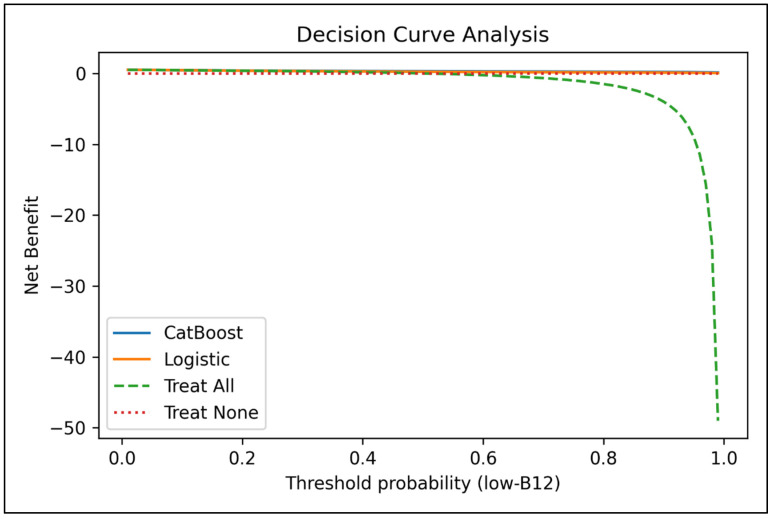
Decision curve analysis of the CatBoost and logistic regression models for predicting vitamin B12 deficiency. The solid blue line represents the CatBoost model, and the solid orange line represents logistic regression. The green dashed line (“Treat All”) assumes all patients would be classified as deficient, whereas the red dotted line (“Treat None”) assumes no intervention. Net benefit is plotted across threshold probabilities for predicting low vitamin B12 levels, demonstrating the clinical utility of each strategy relative to default decision rules.

LIME analyses confirmed the internal consistency of feature contributions for individual-level predictions and reinforced the clinical interpretability of the model (Supplementary Material S1, Figure S1). Decision Curve Analysis (DCA) demonstrated that the model provided a consistently higher net benefit across a wide threshold range compared with “treat-all” or “treat-none” strategies (Figure 4), supporting its potential integration into clinical decision-support systems. In clinical practice, the proposed model is intended to function as a screening and risk-stratification tool within routine laboratory workflows. A positive model prediction is designed to prompt targeted clinical actions, such as prioritization of confirmatory assessment or repeat serum vitamin B12 testing when clinically indicated, rather than immediate therapeutic intervention.

## 4. Discussion

This study represents the first comprehensive machine-learning investigation designed to predict serum vitamin B12 levels directly from routinely collected laboratory measurands. Current literature contains predictive models for vitamin D, folate, lipid metabolism, and other biochemical markers, yet no prior study has focused specifically on estimating B12 status using hematologic and biochemical indices at scale [7,16,17]. By integrating routine laboratory features with explainable artificial intelligence (XAI) techniques, the present work offers a novel and clinically actionable framework that bridges biomedical informatics, laboratory medicine, and nutritional neuroscience.

Across eight machine-learning algorithms evaluated in four sequential experimental settings, gradient-boosting models consistently demonstrated superior ability to capture the nonlinear and population-level variability inherent in biochemical data. Linear models such as logistic regression and support vector machines showed limited generalizability with AUC values around 0.86, consistent with prior work by Sharifmousavi et al. using SVM-based micronutrient prediction in neurological populations [6]. Artificial neural networks yielded lower accuracy (0.78) than the 85% reported by Tamune et al. in B6 deficiency [5], while also suffering from well-recognized limitations in interpretability—an important consideration for clinical deployment. Tree-based ensembles such as random forest similarly demonstrated moderate discrimination but experienced performance instability under external validation, a finding aligned with previously reported limitations of over-partitioning in high-dimensional biomedical data [18].

XGBoost outperformed these baseline models, reflecting its strong capacity to capture interactions between biochemical variables, consistent with prior lipid profiling work (r = 0.98) by Anudeep et al. [8]. However, CatBoost provided the most robust and generalizable performance across all experimental conditions—an observation supported by previous reports emphasizing its unbiased handling of categorical variables and resilience in clinical datasets [19,20]. In our study, CatBoost achieved an AUC-ROC of 0.88, AUC-PR of 0.86, F1 of 0.82, and the most balanced sensitivity–specificity profile across both test and validation cohorts.

Interpretability analyses further strengthened the biological plausibility of the model. SHAP and LIME consistently highlighted MCV, RDW, HGB, HCT, ferritin, CRP, folate, and age as principal contributors to classification. These findings reinforce well-established hematological consequences of B12 deficiency, including macrocytosis, anisocytosis, and impaired erythropoiesis. Elevated contributions of ferritin and CRP mirror the known inhibitory effects of systemic inflammation on circulating B12 bioavailability via haptocorrin-mediated sequestration, as reported by Jensen et al. [21,22]. The observed interplay between folate and B12 also aligns with their shared role in one-carbon metabolism and DNA synthesis [2,17,23]. Age contributed positively, consistent with declining intrinsic factor secretion and reduced gastrointestinal absorption in older adults [24]. Together, these patterns demonstrate that the model not only predicts deficiency but also captures meaningful pathophysiological signatures associated with B12 metabolism.

The sequential experimental framework provided additional insight into how model performance evolved under different analytical strategies. Experiment 1 established baseline performance; Experiment 2 applied F1-maximizing threshold optimization and yielded the most clinically balanced results, increasing sensitivity to 0.92 and reducing false negatives by nearly 45%. Experiment 3 evaluated hyperparameter tuning, which improved discrimination modestly but introduced mild overfitting in the validation cohort—consistent with prior findings on the trade-off between complexity and generalizability in clinical ML models [25]. Experiment 4 introduced feature engineering; however, CatBoost’s intrinsic ability to manage correlated and categorical variables meant that manual engineering added minimal incremental benefit. Collectively, these findings validated Experiment 2 as the optimal configuration for clinical decision support.

When compared with the only available machine-learning study on B12 deficiency, conducted by Tamune et al. in psychiatric patients using a sample of 497 individuals (AUC-ROC 0.62), our model showed substantially higher discrimination (AUC-ROC 0.88) using a dataset nearly 150 times larger, highlighting its superior generalizability and methodological rigor [5]. In contrast to genetic Mendelian randomization studies such as Lu et al. [26], which explore upstream determinants of B12 metabolism, our work emphasizes biochemical phenotypes and their downstream hematologic expression—providing complementary but clinically more immediate insights. Similarly, while Duman et al. demonstrated hematologic alterations in pediatric B12 deficiency [27], their narrower variable set lacked inflammatory and metabolic markers that contributed substantially to our model’s predictive signal.

The proposed AI framework is designed to complement, rather than replace, advanced biomarkers such as holotranscobalamin and methylmalonic acid. By leveraging routinely available hematological and biochemical parameters, the model provides an initial risk stratification layer that may help identify patients who are most likely to benefit from further confirmatory testing. In this respect, the approach supports a stepwise diagnostic strategy, particularly in settings where advanced biomarkers are costly, not routinely available, or reserved for selected cases.

From an implementation perspective, the model has strong potential for integration into laboratory information systems (LIS) or hospital information systems (HIS). Because it relies entirely on existing routine tests (e.g., hemogram, ferritin, CRP, albumin), it imposes no additional cost and can be deployed passively in background systems. LIME-based patient-specific explanations could be displayed to clinicians, allowing early recognition of biochemical patterns suggestive of deficiency before irreversible neurological or hematological complications arise. Early identification of vitamin B12 deficiency may facilitate timely clinical management and help prevent downstream clinical complications. Such a model could therefore contribute to both clinical and economic decision-making. From a patient-centered perspective, the proposed model has the potential to improve diagnostic efficiency by leveraging routinely available laboratory measurands without requiring additional or specialized testing. By supporting earlier and more accurate identification of individuals at risk for vitamin B12 deficiency, the model may help reduce unnecessary repeat measurements or downstream confirmatory tests, thereby lowering laboratory examination expenditure. Earlier diagnosis may also facilitate timely therapeutic intervention, with potential benefits for clinical outcomes and overall healthcare resource utilization.

### Limitations

This study has several limitations. Its retrospective nature prevented inclusion of symptoms, dietary intake, medication history, gastrointestinal disorders, and genetic factors—variables known to influence B12 metabolism. Only adult patients were included, limiting generalizability to pediatric populations. Although data were obtained from the laboratory information system, all results had undergone biochemical specialist approval, ensuring that pre-examination artifacts such as hemolysis or improper storage were already filtered out. Missing values were removed rather than imputed, eliminating imputation bias but introducing a theoretical selection bias. Classification thresholds were based on the NICE 2024 guideline, ensuring standardization across laboratories [11]. Nevertheless, total serum B12 is an imperfect marker of functional deficiency; lack of holo-transcobalamin or methylmalonic acid data may lead to misclassification in certain subgroups [28]. Finally, micronutrient patterns may vary across regions and populations, suggesting that model recalibration may be necessary before widespread deployment [29]. As with all clinical ML models, temporal performance drift remains a potential risk and requires periodic re-evaluation [30]. Although the present model was developed using data from a single tertiary-care center, it relies exclusively on routinely available laboratory analytes and reference-based encoding schemes that are widely standardized across clinical laboratories. This design enhances its potential transferability to institutions using comparable analytical platforms and quality-control procedures. The use of a fully independent temporal validation cohort further supports real-world generalizability across time. Nevertheless, external multicenter validation and site-specific recalibration will be required before widespread clinical deployment. Serum total vitamin B12 levels were not measured using the gold-standard isotope-dilution liquid chromatography–tandem mass spectrometry (ID-LC–MS/MS) method. Instead, a chemiluminescence immunoassay was used, which is subject to method-dependent analytical bias, including variability in antibody specificity and differential detection of protein-bound B12. Therefore, assay-related misclassification cannot be fully excluded. Accordingly, the model should be interpreted as a screening and risk-stratification tool for laboratory-defined vitamin B12 deficiency rather than a definitive diagnostic instrument for functional cobalamin deficiency. Therefore, predictive values and net clinical benefit estimates may differ if the model is applied in population-level screening settings with lower prevalence of vitamin B12 deficiency.

## 5. Conclusions

Despite these limitations, this study introduces the first large-scale, explainable, and clinically validated machine-learning model for predicting vitamin B12 deficiency using routine laboratory measurands. The model demonstrates high discrimination, strong biological interpretability, and stable performance across internal and temporal validation cohorts. When integrated into clinical workflows, it has the potential to support early diagnosis, reduce unnecessary testing, and enhance decision-making in nutritional and hematologic assessments. Future work should focus on multi-center prospective validation, continuous monitoring for model drift, and extension of the framework to other micronutrient deficiencies such as folate, vitamin D, and ferritin. In parallel, evaluating how this explainable AI model behaves when quietly embedded into routine laboratory workflows—augmenting clinical decisions without disrupting established pathways—will be essential for realizing its full translational impact.

## Figures and Tables

**Table 1:** Descriptive Statistics and Effect Size (Cohen’s d) for All Variables.

Variable	Unit	Development Dataset Mean ± SD	Validation Dataset Mean ± SD	*p*-Value	Cohen d (Effect)
Age	years	49.43 ± 16.66	41.42 ± 24.99	<0.05	0.377
B12	pg/mL	277.27 ± 203.78	308.62 ± 234.34	<0.05	−0.143
Folate	ng/mL	8.3 ± 3.98	8.97 ± 4.51	<0.05	0.197
Glucose	mg/dL	99.71 ± 38.00	99.65 ± 45.98	0.85	0.001
ALT	U/L	22.83 ± 26.05	26.12 ± 75.04	<0.05	−0.059
AST	U/L	22.35 ± 21.01	30.39 ± 206.21	<0.05	−0.055
Ferritin	ng/mL	236.00 ± 987.00	99.99 ± 204.05	<0.05	0.191
ALP	U/L	72.48 ± 36.35	112.84 ± 97.94	<0.05	−0.546
GGT	U/L	26.41 ± 40.01	33.10 ± 81.44	<0.05	−0.104
T. Bilirubin	mg/dL	0.69 ± 0.34	0.78 ± 0.93	<0.05	−0.129
Creatinine	mg/dL	0.88 ± 0.36	0.95 ± 0.85	<0.05	−0.114
C-RP	mg/L	6.55 ± 16.10	10.92 ± 29.11	<0.05	−0.186
Albumin	g/L	43.60 ± 3.86	43.21 ± 6.23	<0.05	0.074
T. Protein	g/L	72.83 ± 4.90	72.02 ± 6.12	<0.05	0.145
Iron	µg/dL	176.51 ± 148.63	176.32 ± 153.23	0.87	0.001
LDH	U/L	176.47 ± 61.25	208.16 ± 215.55	<0.05	−0.200
FT4	ng/dL	0.89 ± 0.17	0.92 ± 0.20	<0.05	−0.137
TSH	µIU/mL	2.18 ± 2.46	2.29 ± 2.58	<0.05	−0.045
HGB	g/dL	13.43 ± 1.67	12.84 ± 1.91	<0.05	0.331
RBC	10^6^/µL	4.69 ± 0.53	4.61 ± 0.64	<0.05	0.133
HCT	%	40.19 ± 4.44	38.75 ± 5.18	<0.05	0.298
MCV	fL	85.89 ± 6.28	84.62 ± 7.78	<0.05	0.179
MCH	pg	28.76 ± 2.60	28.17 ± 3.07	<0.05	0.208
MCHC	g/dL	33.48 ± 1.24	33.32 ± 1.34	<0.05	0.123
RDW	%	14.03 ± 1.80	14.41 ± 2.52	<0.05	−0.174
WBC	10^3^/µL	7.26 ± 2.25	8.20 ± 6.79	<0.05	−0.186
NEU	10^3^/µL	5.50 ± 1.44	4.07 ± 3.37	<0.05	0.552
LYM	10^3^/µL	3.08 ± 0.98	2.46 ± 5.43	<0.05	0.159
MONO	10^3^/µL	0.79 ± 0.23	0.64 ± 0.49	<0.05	0.382
BASO	10^3^/µL	0.64 ± 0.46	0.05 ± 0.21	<0.05	1.641
PLT	10^3^/µL	269.78 ± 75.84	284.84 ± 106.67	<0.05	−0.163
MPV	fL	9.49 ± 1.27	9.73 ± 1.33	<0.05	−0.183
PDW	fL	15.27 ± 2.54	14.22 ± 3.00	<0.05	0.376

SD: standart deviation.

**Table 2:** Performance comparison of eight machine-learning models under the F1-max threshold (experiment 2).

Metrics/Model	CatBoost	XGB	ANN	RF	LR	SVM	KNN	DT
CutOff	0.402	0.378	0.372	0.410	0.396	0.306	0.400	0.500
TP	3233	3265	3268	3184	3098	3216	3297	2468
FP	1160	1198	1325	1197	1452	2006	2473	994
TN	2368	2330	2203	2331	2076	1522	1055	2534
FN	295	263	260	344	430	312	231	1060
Sensitivity	0.92 (90.6–92.53)	0.92 (91.6–93.4)	0.93 (91.7–93.4)	0.90 (89.2–91.2)	0.87 (86.7–88.9)	0.91 (90.2–92.0)	0.93 (92.6–94.2)	0.70 (68.4–71.4)
Specificity	0.67 (65.5–68.7)	0.66 (64.5–67.6)	0.62 (60.8–64.0)	0.66 (64.5–67.6)	0.59 (57.2–60.5)	0.43 (41.5–44.8)	0.30 (28.4–31.4)	0.72 (70.3–73.3)
PPV	0.73 (72.7–74.5)	0.73 (71.8–74.4)	0.71 (69.8–72.4)	0.73 (71.3–74.0)	0.68 (66.7–69.4)	0.61 (60.3–62.9)	0.57 (55.9–58.4)	0.71 (69.8–72.8)
NPV	0.88 (87.8–89.0)	0.89 (88.6–91.0)	0.89 (88.2–90.6)	0.87 (85.8–88.4)	0.82 (81.3–84.3)	0.83 (81.2–84.6)	0.82 (79.8–84.0)	0.70 (69.0–72.0)
PLR	2.79 (2.66–2.92)	2.73 (2.60–2.86)	2.47 (2.36–2.58)	2.66 (2.54–2.79)	2.13 (2.05–2.22)	1.60 (1.55–1.65)	1.33 (1.30–1.36)	2.48 (2.35–2.63)
NLR	0.12 (3.13–3.52)	0.11 (0.10–0.13)	0.12 (0.10–0.13)	0.15 (0.13–0.16)	0.21 (0.19–0.23)	0.20 (0.18–0.23)	0.22 (0.19–0.25)	0.42 (0.40–0.44)
F1	0.82 (0.81–0.83)	0.82 (0.81–0.83)	0.80 (0.80–0.81)	0.81 (0.80–0.81)	0.77 (0.76–0.78)	0.74 (0.72–0.75)	0.71 (0.70–0.72)	0.71 (0.69–0.72)
Accuracy	0.79 (78.4–80.3)	0.79 (78.3–80.2)	0.78 (76.5–78.5)	0.78 (77.2–79.1)	0.73 (72.3–74.3)	0.67 (66.0–68.2)	0.62 (60.5–62.8)	0.71 (69.8–71.9)
A_ROC	0.88 (0.86–0.90)	0.88 (0.87–0.89)	0.86 (0.85–0.87)	0.86 (0.85–0.87)	0.81 (0.79–0.82)	0.77 (0.76–0.78)	0.72 (0.70–0.73)	0.71 (0.70–0.72)
A_PR	0.86 (0.84–0.88)	0.85 (0.84–0.86)	0.84 (0.82–0.85)	0.82 (0.80–0.83)	0.78 (0.76–0.79)	0.75 (0.74–0.77)	0.66 (0.65–0.68)	0.65 (0.63–0.66)
MCC	0.61 (0.58–0.62)	0.61 (0.58–0.62)	0.58 (0.55–0.59)	0.58 (0.56–0.6)	0.49 (0.47–0.50)	0.39 (0.37–0.41)	0.30 (0.28–0.32)	0.42 (0.39–0.43)

CatBoost: Categorical Gradient Boosting; XGB: Extreme Gradient Boosting (XGBoost); ANN: Artificial Neural Network; RF: Random Forest; LR: Logistic Regression; SVM: Support Vector Machine; KNN: k-Nearest Neighbors; DT: Decision Tree; TP: true positive; FP: false positive; TN: true negative; FN: false negative; PPV: positive predictive value; NPV: negative predictive value; PLR: positive likelihood ratio; NLR: negative likelihood ratio; A_ROC: area under the ROC curve; A_PR: area under the precision–recall curve; MCC: Matthews correlation coefficient.

**Table 3:** Performance Metrics of the CatBoost Model Across All Experimental Configurations.

Performance Metrics	Experiment 1 (Default Parameter)	Experiment 2 (Cut-Off Optimization)	Experiment 3 (Hyperparameter Tuning)	Experiment 4 (Feature Engineering)
TP	2993	3233	3028	3014
FP	895	1160	924	908
TN	2633	2368	2604	2620
FN	535	295	500	514
Sensitivity	0.85 (0.84–0.86)	0.92 (0.91–0.93)	0.86 (0.85–0.87)	0.85 (0.84–0.86)
Specificity	0.75 (0.73–0.76)	0.67 (0.65–0.69)	0.74 (0.73–0.75)	0.74 (0.73–0.75)
PPV	0.77 (0.76–0.78)	0.73 (0.72–0.75)	0.77 (0.76–0.78)	0.77 (0.76–0.78)
NPV	0.83 (0.82–0.84)	0.88 (0.87–0.89)	0.84 (0.83–0.85)	0.84 (0.83–0.85)
PLR	3.34 (3.15–3.55)	2.79 (2.65–2.94)	3.28 (3.12–3.44)	3.32 (3.24–3.40)
NLR	0.20 (0.19–0.22)	0.12 (0.10–0.14)	0.19 (0.18–0.20)	0.20 (0.20–0.20)
Accuracy	0.81 (0.80–0.82)	0.79 (0.78–0.80)	0.80 (0.79–0.81)	0.80 (0.79–0.81)
F1	0.80 (0.79–0.81)	0.82 (0.81–0.83)	0.81 (0.80–0.82)	0.81 (0.80–0.82)
AUC-ROC	0.88 (0.87–0.89)	0.88 (0.87–0.89)	0.88 (0.87–0.89)	0.86 (0.85–0.87)
AUC-PR	0.86 (0.85–0.87)	0.86 (0.85–0.87)	0.86 (0.85–0.87)	0.88 (0.87–0.89)
MCC	0.60 (0.58–0.62)	0.61 (0.59–0.63)	0.60 (0.58–0.62)	0.60 (0.59–0.61)

TP: true positive; FP: false positive; TN: true negative; FN: false negative; PPV: positive predictive value; NPV: negative predictive value; PLR: positive likelihood ratio; NLR: negative likelihood ratio; AUC-ROC: area under the ROC curve; AUC-PR: area under the precision–recall curve; MCC: Matthews correlation coefficient.

**Table 4:** Performance of the Validation Set (Final CatBoost Model).

Performance Metric	Validation Set
TP	15,018
FP	3910
TN	1320
FN	2576
Sensitivity	0.85 (0.84–0.86)
Specificity	0.77 (0.75–0.79)
PPV	0.79 (0.78–0.80)
NPV	0.83 (0.82–0.84)
PLR	3.74 (3.61–3.87)
NLR	0.19 (0.18–0.20)
Accuracy	0.81 (0.80–0.82)
F1 Score	0.82 (0.81–0.83)
AUC-ROC	0.90 (0.89–0.91)
AUC-PR	0.91 (0.90–0.92)
MCC	0.63 (0.61–0.65)

TP: true positive; FP: false positive; TN: true negative; FN: false negative; PPV: positive predictive value; NPV: negative predictive value; PLR: positive likelihood ratio; NLR: negative likelihood ratio; AUC-ROC: area under the ROC curve; AUC-PR: area under the precision–recall curve; MCC: Matthews correlation coefficient.

## Data Availability

The data presented in this study are not publicly available due to institutional regulations and patient privacy considerations. Aggregated data supporting the findings of this study are available from the corresponding author upon reasonable request and with appropriate ethical approval.

## Supplementary Materials

The following supporting information can be downloaded at: https://www.mdpi.com/article/10.3390/diagnostics16040563/s1, Supplementary Material S1: Table S1: Thresholds Used for Laboratory Variable Categorization; Table S2: Distribution of Variables by Age and Sex in the Development Cohort and Descriptive Statistics; Table S3: Distribution of Variables by Age and Sex in the Validation Cohort and Descriptive Statistics; Table S4: Performance of baseline machine-learning models evaluated in Experiment 1; Table S5: Results of Experiment 3, demonstrating model performance after hyperparameter optimization; Table S6: Results of Experiment 4 assessing the impact of feature engineering strategies on model performance; Figure S1: Localinterpretable model-agnostic explanation (LIME) plot showing feature-level contributions for a single prediction made by the CatBoost classifier. The following Supplementary Materials are available online in Supplementary Material S2: Sheet 1: Feature Engineering Rules.
