# Supplementary material for: Development and Temporal Validation of Explainable Machine Learning Models for Predicting Vitamin B12 Deficiency Using Routine Laboratory Analytes
Source: Diagnostics (Basel). 2026 Feb 13;16(4):563. doi: 10.3390/diagnostics16040563 (PMC12939089; doi:10.3390/diagnostics16040563)
Supplement: Supplementary file 1 [file diagnostics-16-00563-s001.zip › Supplementary Material - S1.pdf]

SM S1 Table S1. Thresholds Used for Laboratory Variable Categorization

No	Variable	Unit	Men – 1 Lower	Men – 1 Upper	Women – 2 Lower	Women – 2 Upper	Reference
1	B12	pg/mL	350,00	900	350,00	900	NICE guideline 2024
2	Glucose	mg/dL	70,00	100,00	70,00	100,00	DiabetesResClinPract. 2025 Aug;226:112323.
3	Folate	ng/mL	2,00	20,00	2,00	20,00	Manual of LaboratoryandDiagnosticTests. 9th ed. Philadelphia: Lippincott
4	ALT	U/L	0,00	50,00	0,00	50,00	RRF
5	AST	U/L	0,00	50,00	0,00	50,00	RRF
6	Ferritin	ng/mL	15,00	200,00	15,00	150,00	WHO guideline on use of ferritin
7	FT4	ng/dL	0,93	1,71	0,93	1,71	GuidelinefortheDiagnosisandTreatment of ThyroidDiseasesby TEMD
8	TSH	µIU/mL	0,27	4,20	0,27	4,20	
9	ALP	U/L	30,00	120,00	30,00	120,00	RRF
10	GGT	U/L	0,00	55,00	0,00	55,00	RRF
11	T. Bilirubin	mg/dL	0,00	0,20	0,00	0,20	RRF
12	Creatinine	mg/dL	0,60	1,20	0,50	1,10	Mosby'sDiagnostic&Laboratory Test Reference. 14th ed.
13	C-RP	mg/L	0,00	5,00	0,00	5,00	RRF
14	Albumin	g/L	35,00	52,00	35,00	52,00	RRF
15	T. Protein	g/L	66,00	83,00	66,00	83,00	RRF
16	Iron	µg/dL	80,00	180,00	60,00	160,00	Biochemistry, IronAbsorption - NBK448204
17	LDH	U/L	0,00	248,00	0,00	248,00	RRF
18	HGB	g/dL	13,00	16,50	12,00	16,00	WHO Guideline on haemoglobincutoffs …
19	NEU	$10^3/\mu L$	1,78	5,38	1,78	5,38	RRF
20	MPV	fL	9,40	12,40	9,40	12,40	RRF
21	LYM	$10^3/\mu L$	1,00	4,80	1,00	4,80	RRF
22	MONO	$10^3/\mu L$	0,19	0,77	0,19	0,77	RRF
23	WBC	$10^3/\mu L$	3,91	8,77	3,91	8,77	RRF
24	BASO	$10^3/\mu L$	0,01	0,05	0,01	0,05	RRF
25	RBC	$10^6/\mu L$	4,18	5,48	4,18	5,48	RRF
26	MCH	pg	26,00	36,00	26,00	36,00	RRF
27	MCV	fL	80,00	100,00	80,00	100,00	RRF
28	MCHC	g/dL	32,00	36,00	32,00	36,00	RRF
29	PDW	fL	10,10	16,10	10,10	16,10	RRF
30	PLT	$10^3/\mu L$	151,00	304,00	151,00	304,00	RRF
31	HCT	%	41,00	49,00	36,00	48,00	WHO Guideline on haemoglobincutoffs …
32	RDW	%	11,50	14,50	11,50	14,50	RRF

RRF: recommended reference intervals provided by the assay manufacturers and recommended for routine clinical use in Türkiye.

SM S1 Table S2. Distribution of Variables by Age and Sex in the Development Cohort and Descriptive Statistics

	**Men**			**Women**			**Gender**	**Age - men**	**Age - women**
**Variables**	**18–39**<br>n: 3459	**40–64**<br>n: 5442	**≥65**<br>n: 5207	**18–39**<br>n: 6151	**40–64**<br>n: 8674	**≥65**<br>n: 6343	**p**	**p**	**p**
Age	26.93 ± 6.98	53.33 ± 7.05	74.73 ± 7.16	28.00 ± 6.73	51.90 ± 7.16	75.41 ± 7.62	0.000	0.000	0.000
B12	244.73 ± 176.77	290.86 ± 240.99	353.10 ± 313.28	252.52 ± 177.79	294.79 ± 224.46	395.52 ± 345.91	0.000	0.000	0.000
Glucose	90.04 ± 30.06	112.33 ± 56.12	119.13 ± 58.09	87.53 ± 28.62	102.44 ± 42.77	119.15 ± 58.48	0.000	0.000	0.000
Folate	7.43 ± 3.85	8.27 ± 4.06	8.36 ± 4.63	8.26 ± 4.35	9.34 ± 4.50	9.76 ± 5.08	0.000	0.000	0.000
ALT	37.32 ± 118.86	35.81 ± 68.51	32.16 ± 124.56	21.75 ± 46.92	26.02 ± 70.37	24.43 ± 54.44	0.000	**0.055**	0.000
AST	31.65 ± 123.28	30.84 ± 49.86	36.71 ± 157.93	22.36 ± 37.76	25.78 ± 104.91	30.95 ± 125.65	0.000	0.025	0.000
Ferritin	231.20 ± 94.33	192.50 ± 78.10	296.83 ± 98.78	495.42 ± 141.13	494.01 ± 139.56	375.72 ± 115.73	0.000	0.000	0.000
FT4	0.91 ± 0.17	0.92 ± 0.22	0.99 ± 0.24	0.88 ± 0.19	0.90 ± 0.21	1.01 ± 0.25	0.000	0.000	0.000
TSH	2.05 ± 1.77	2.07 ± 2.97	1.94 ± 3.22	2.19 ± 2.41	2.45 ± 2.95	2.42 ± 3.60	0.000	0.040	0.000
ALP	102.66 ± 78.69	89.93 ± 75.84	101.76 ± 121.94	69.09 ± 44.13	79.18 ± 54.50	91.74 ± 84.18	0.000	0.000	0.000
GGT	38.63 ± 82.45	62.33 ± 143.83	61.90 ± 145.96	20.64 ± 43.96	32.20 ± 75.20	41.47 ± 93.57	0.000	0.000	0.000
Total Bilirubin	0.80 ± 1.03	0.98 ± 1.46	1.13 ± 1.76	0.68 ± 0.76	0.71 ± 0.69	0.89 ± 1.06	0.000	0.000	0.000
Creatinine	1.00 ± 0.83	1.25 ± 1.27	1.51 ± 1.31	0.77 ± 0.51	0.89 ± 0.73	1.22 ± 1.00	0.000	0.000	0.000
C-Reactive Protein	6.28 ± 18.12	16.98 ± 39.08	30.40 ± 53.20	5.17 ± 15.82	10.25 ± 26.85	21.86 ± 42.30	0.000	0.000	0.000
Albumin	45.57 ± 4.58	41.94 ± 5.64	38.42 ± 6.22	44.09 ± 4.00	42.25 ± 4.18	39.15 ± 5.59	0.000	0.000	0.000
Total Protein	72.38 ± 6.07	71.31 ± 6.94	68.52 ± 7.69	72.62 ± 5.41	72.06 ± 5.72	69.02 ± 7.40	0.000	0.000	0.000
Iron	194.27 ± 149.40	168.06 ± 142.19	146.04 ± 134.18	204.01 ± 165.46	171.94 ± 154.04	143.58 ± 138.10	0.000	0.000	0.000
LDH	196.06 ± 179.63	226.11 ± 318.37	260.39 ± 552.71	173.14 ± 79.47	198.81 ± 113.36	241.67 ± 252.10	0.000	0.000	0.000
HGB	14.56 ± 1.89	13.92 ± 2.28	12.39 ± 2.36	12.48 ± 1.42	12.49 ± 1.60	11.73 ± 1.88	0.000	0.000	0.000
NEU	54.38 ± 17.09	55.08 ± 21.50	58.75 ± 24.28	55.82 ± 15.74	55.25 ± 17.66	57.62 ± 22.21	**0.529**	0.000	0.000
MPV	9.08 ± 1.21	9.19 ± 1.24	9.28 ± 1.29	9.30 ± 1.26	9.35 ± 1.26	9.47 ± 1.32	0.000	0.000	0.000
LYM	2.98 ± 1.14	2.75 ± 1.37	2.39 ± 1.51	2.99 ± 1.05	2.96 ± 1.14	2.64 ± 1.42	0.000	0.000	0.000
MONO	0.86 ± 0.34	0.85 ± 0.34	0.91 ± 0.47	0.79 ± 0.29	0.78 ± 0.30	0.84 ± 0.35	0.000	0.000	0.000
WBC	7.56 ± 2.62	8.80 ± 8.17	9.65 ± 13.49	7.30 ± 2.67	7.67 ± 6.27	8.76 ± 13.23	0.000	0.000	0.000
BASO	0.58 ± 0.45	0.73 ± 1.37	0.79 ± 0.83	0.58 ± 0.42	0.68 ± 0.54	0.80 ± 0.85	0.000	0.000	0.000
RBC	5.10 ± 0.62	4.78 ± 0.76	4.33 ± 0.78	4.48 ± 0.43	4.44 ± 0.54	4.17 ± 0.67	0.000	0.000	0.000
MCH	28.75 ± 2.69	29.65 ± 3.22	29.58 ± 3.56	28.07 ± 2.94	28.49 ± 3.30	29.01 ± 3.50	0.000	0.000	0.000
MCV	84.59 ± 6.40	87.36 ± 7.48	88.22 ± 8.64	83.97 ± 7.07	85.34 ± 8.17	87.21 ± 8.48	0.000	0.000	0.000
MCHC	34.00 ± 1.17	34.02 ± 1.31	33.70 ± 1.39	33.41 ± 1.21	33.39 ± 1.24	33.37 ± 1.38	0.000	0.000	**0.317**
PDW	15.82 ± 2.29	15.96 ± 2.45	16.44 ± 2.38	15.91 ± 2.15	15.92 ± 2.26	16.13 ± 2.39	0.000	0.000	0.000
PLT	265.15 ± 102.99	256.67 ± 111.1	247.74 ± 124.68	275.80 ± 89.60	279.55 ± 112.14	271.89 ± 128.65	0.000	0.000	0.000
HCT	42.97 ± 5.07	41.40 ± 6.07	37.65 ± 6.39	37.45 ± 3.75	37.60 ± 4.33	35.90 ± 5.29	0.000	0.000	0.000
RDW	14.08 ± 2.29	14.71 ± 2.96	16.03 ± 3.49	14.48 ± 2.50	14.91 ± 2.84	15.70 ± 3.20	**0.580**	0.000	0.000

p-men reflects within- male age-group differences; p-women reflects within- female age-group differences; p-gender tests sex-based age differences within each age category. SD: standard deviation

SM S1 Table S3. Distribution of Variables by Age and Sex in the Validation Cohort and Descriptive Statistics

**Variable**	**Men**			**Women**			**p–men**	**p–wo men**	**p-Gen der**
	**18–39**	**40–64**	**≥65**	**18–39**	**40–64**	**≥65**			
	n:4070	n:5610	n:4855	n:6043	n:7936	n:6230			
Age	21.44 ± 10.64	41.99 ± 20.08	55.12 ± 29.87	23.50 ± 10.16	43.45 ± 17.96	58.07 ± 28.93	0.000	0.000	0.000
B12	268.20 ± 173.48	296.55 ± 224.76	338.68 ± 270.56	265.82 ± 169.87	302.59 ± 215.73	371.65 ± 298.54	0.000	0.000	0.000
Glucose	88.09 ± 29.00	106.28 ± 53.88	107.82 ± 51.08	85.65 ± 28.23	99.33 ± 44.09	108.86 ± 53.77	0.000	0.000	0.000
Folate	8.52 ± 4.68	8.95 ± 4.52	9.17 ± 5.00	8.78 ± 4.76	9.50 ± 4.69	10.00 ± 5.21	0.000	0.000	0.000
ALT	35.09 ± 110.13	30.11 ± 50.71	25.57 ± 81.55	21.04 ± 47.65	24.81 ± 69.42	23.70 ± 86.88	0.000	0.006	0.000
AST	33.86 ± 112.60	36.37 ± 456.95	30.58 ± 45.74	24.89 ± 48.18	27.20 ± 111.07	31.99 ± 145.57	**0.598**	0.001	0.000
Ferritin	105.14 ± 184.75	146.33 ± 234.77	148.00 ± 241.55	40.57 ± 89.36	71.36 ± 187.57	111.60 ± 232.21	0.000	0.000	0.000
FT4	0.91 ± 0.16	0.90 ± 0.19	0.95 ± 0.21	0.88 ± 0.18	0.89 ± 0.20	0.96 ± 0.23	0.000	0.000	0.000
TSH	2.21 ± 1.75	2.15 ± 2.54	2.07 ± 2.11	2.23 ± 1.89	2.49 ± 2.87	2.47 ± 3.43	0.011	0.000	0.000
HGB	14.10 ± 1.96	13.73 ± 2.14	12.68 ± 2.12	12.50 ± 1.34	12.54 ± 1.52	12.03 ± 1.71	0.000	0.000	0.000
NEU	3.75 ± 2.03	4.15 ± 3.28	4.50 ± 5.07	3.88 ± 2.03	3.95 ± 3.43	4.21 ± 3.43	0.000	0.000	0.001
MPV	9.52 ± 1.28	9.61 ± 1.30	9.68 ± 1.37	9.74 ± 1.32	9.81 ± 1.31	9.89 ± 1.36	0.000	0.000	0.000
LYM	2.46 ± 1.24	2.56 ± 5.04	2.63 ± 9.26	2.30 ± 1.06	2.35 ± 3.13	2.54 ± 7.77	**0.440**	0.011	0.000
MONO	0.65 ± 0.25	0.69 ± 0.46	0.75 ± 0.75	0.58 ± 0.22	0.58 ± 0.40	0.66 ± 0.63	0.000	0.000	0.000
WBC	7.91 ± 2.66	8.57 ± 6.19	9.05 ±11.2	7.64 ± 2.50	7.80 ± 5.12	8.46 ± 8.85	0.000	0.000	0.000
BASO	0.05 ± 0.03	0.06 ± 0.12	0.07 ± 0.50	0.04 ± 0.03	0.05 ± 0.15	0.06 ± 0.08	0.005	0.000	0.000
RBC	5.07 ± 0.58	4.84 ± 0.68	4.53 ± 0.75	4.56 ± 0.42	4.52 ± 0.51	4.34 ± 0.64	0.000	0.000	0.000
MCH	27.94 ± 2.70	28.75 ± 3.19	28.55 ± 3.32	27.57 ± 2.76	28.00 ± 3.10	28.30 ± 3.12	0.000	0.000	0.000
MCV	82.84 ± 6.61	85.30 ± 7.74	85.64 ± 8.70	83.25 ± 6.82	84.58 ± 7.74	85.77 ± 8.26	0.000	0.000	0.000
MCHC	33.72 ± 1.17	33.75 ± 1.39	33.43 ± 1.36	33.10 ± 1.26	33.10 ± 1.30	33.09 ± 1.33	0.000	**0.799**	0.000
PDW	13.91 ± 2.98	14.17 ± 3.09	14.41 ± 3.19	14.19 ± 2.87	14.28 ± 2.89	14.30 ± 3.05	0.000	**0.073**	0.000
PLT	284.64 ± 99.97	275.16 ± 109.36	269.41 ± 115.59	294.07 ± 89.64	290.65 ± 104.61	289.37 ± 116.62	0.000	0.035	0.000
HCT	41.88 ± 5.31	41.02 ± 5.82	38.37 ± 5.85	37.81 ± 3.59	38.03 ± 4.14	36.80 ± 4.82	0.000	0.000	0.000
RDW	13.74 ± 1.98	14.22 ± 2.46	15.05 ± 3.06	14.12 ± 2.20	14.42 ± 2.49	14.81 ± 2.60	0.000	0.000	0.000
ALP	133.00 ± 110.37	119.18 ± 100.00	131.24 ± 114.02	97.34 ± 94.28	98.99 ± 79.63	112.29 ± 93.08	0.000	0.000	0.000
GGT	31.76 ± 66.38	48.74 ± 121.17	43.24 ± 102.25	18.97 ± 33.38	28.45 ± 72.31	31.64 ± 66.94	0.000	0.000	0.000
Total Bilirubin	0.85 ± 0.89	0.88 ± 1.29	0.93 ± 1.24	0.66 ± 0.55	0.69 ± 0.67	0.77 ± 0.83	0.003	0.000	0.000
Creatinine	0.88 ± 0.70	1.09 ± 1.10	1.20 ± 1.06	0.73 ± 0.46	0.84 ± 0.67	1.05 ± 0.90	0.000	0.000	0.000
CRP	6.64 ± 21.13	13.38 ± 34.50	19.23 ± 41.94	4.94 ± 13.45	8.15 ± 22.01	14.38 ± 32.77	0.000	0.000	0.000
Albumin	45.67 ± 4.17	43.23 ± 5.18	41.35 ± 5.92	44.61 ± 3.65	43.32 ± 4.10	41.56 ± 10.4	0.000	0.000	0.000
Total Protein	73.42 ± 5.80	71.92 ± 6.47	70.41 ± 6.93	73.16 ± 5.14	72.45 ± 5.32	70.82 ± 6.65	0.000	0.000	0.000
Iron	188.97 ± 150.29	176.50 ± 149.12	161.63 ± 143.45	203.46 ± 165.39	176.68 ± 156.26	152.56 ± 144.77	0.000	0.000	0.000
LDH	204.83 ± 160.59	215.98 ± 208.10	232.66 ± 395.01	179.10 ± 81.97	197.02 ± 113.17	226.55 ± 238.83	0.000	0.000	0.000

p-men reflectswithin-maleage-groupdifferences; p-womenreflectswithin-femaleage-groupdifferences; p-gendertestssex-basedagedifferenceswithineachagecategory. SD: standart deviation

SM Table S4– Performance of baseline machine-learning models evaluated in Experiment 1

Model	Sensitivity	Specificity	PPV	NPV	F1	AUC-ROC	AUC-PR	MCC
CatBoost	0.85	0.75	0.77	0.83	0.81	0.88	0.86	0.6
XGBoost	0.85	0.74	0.77	0.83	0.8	0.88	0.85	0.59
ANN	0.77	0.78	0.78	0.77	0.77	0.86	0.84	0.53
RF	0.84	0.72	0.75	0.82	0.79	0.86	0.82	0.56
LR	0.76	0.71	0.72	0.75	0.74	0.81	0.78	0.46
SVM	0.72	0.66	0.68	0.71	0.7	0.77	0.75	0.38
KNN	0.79	0.53	0.62	0.71	0.7	0.72	0.66	0.31
DT	0.7	0.72	0.71	0.7	0.7	0.71	0.65	0.36

Sensitivity: true positive rate; Specificity: true negative rate; PPV: positive predictive value; NPV: negative predictive value; F1: harmonic mean of precision and recall; AUC-ROC: area under the receiver operating characteristic curve; AUC-PR: area under the precision–recall curve; MCC: Matthews correlation coefficient; ANN: artificial neural network; RF: random forest; LR: logistic regression; SVM: support vector machine; KNN: k-nearest neighbors; DT: decision tree.

SM Table S5 – Results of Experiment 3, demonstrating model performance after hyperparameter optimization.

Model	Sensitivity	Specificity	PPV	NPV	F1	AUC-ROC	AUC-PR	MCC
CatBoost	0.86	0.74	0.77	0.84	0.81	0.88	0.86	0.6
XGBoost	0.87	0.72	0.76	0.85	0.81	0.88	0.85	0.6
ANN	0.77	0.69	0.74	0.77	0.77	0.86	0.83	0.57
RF	0.83	0.73	0.75	0.81	0.79	0.86	0.82	0.56
LR	0.76	0.71	0.72	0.75	0.75	0.81	0.78	0.47
SVM	0.79	0.68	0.71	0.76	0.75	0.81	0.78	0.47
KNN	0.79	0.53	0.63	0.72	0.77	0.72	0.67	0.33
DT	0.93	0.51	0.65	0.87	0.77	0.76	0.68	0.48

Hyperparameters were tuned using GridSearchCV, RandomizedSearchCV, and Optuna-based Bayesian optimization. The table summarizes the optimized hyperparameter configurations and corresponding performance metrics for each machine-learning model evaluated on the validation/test dataset

Sensitivity: true positive rate; Specificity: true negative rate; PPV: positive predictive value; NPV: negative predictive value; F1: harmonic mean of precision and recall; AUC-ROC: area under the receiver operating characteristic curve; AUC-PR: area under the precision–recall curve; MCC: Matthews correlation coefficient; ANN: artificial neural network; RF: random forest; LR: logistic regression; SVM: support vector machine; KNN: k-nearest neighbors; DT: decision tree.

.

SM Table S6. Results of Experiment 4 assessing the impact of feature engineering strategies on model performance.

Model	Sensitivity	Specificity	PPV	NPV	F1	AUC-ROC	AUC-PR	MCC
CatBoost	0.85	0.74	0.77	0.84	0.81	0.88	0.86	0.6
XGBoost	0.84	0.75	0.77	0.82	0.8	0.88	0.85	0.59
SVM	0.84	0.73	0.76	0.82	0.8	0.86	0.83	0.58
ANN	0.74	0.73	0.73	0.74	0.74	0.82	0.78	0.47
KNN	0.85	0.62	0.69	0.81	0.76	0.81	0.77	0.48
RF	0.82	0.73	0.75	0.8	0.76	0.81	0.77	0.55
DT	0.7	0.7	0.7	0.7	0.7	0.7	0.64	0.4
LR	0.83	0.72	0.75	0.81	0.79	0.86	0.84	0.55

The table compares classification metrics obtained using different feature sets, including original laboratory parameters and engineered features. Performance metrics are reported to illustrate the effect of feature engineering on predictive accuracy and overall model robustness.

Sensitivity: true positive rate; Specificity: true negative rate; PPV: positive predictive value; NPV: negative predictive value; F1: harmonic mean of precision and recall; AUC-ROC: area under the receiver operating characteristic curve; AUC-PR: area under the precision–recall curve; MCC: Matthews correlation coefficient; ANN: artificial neural network; RF: random forest; LR: logistic regression; SVM: support vector machine; KNN: k-nearest neighbors; DT: decision tree.

SM S1 Figure S1. Localinterpretable model-agnostic explanation (LIME) plot showing feature-level contributions for a single prediction made by the CatBoost classifier

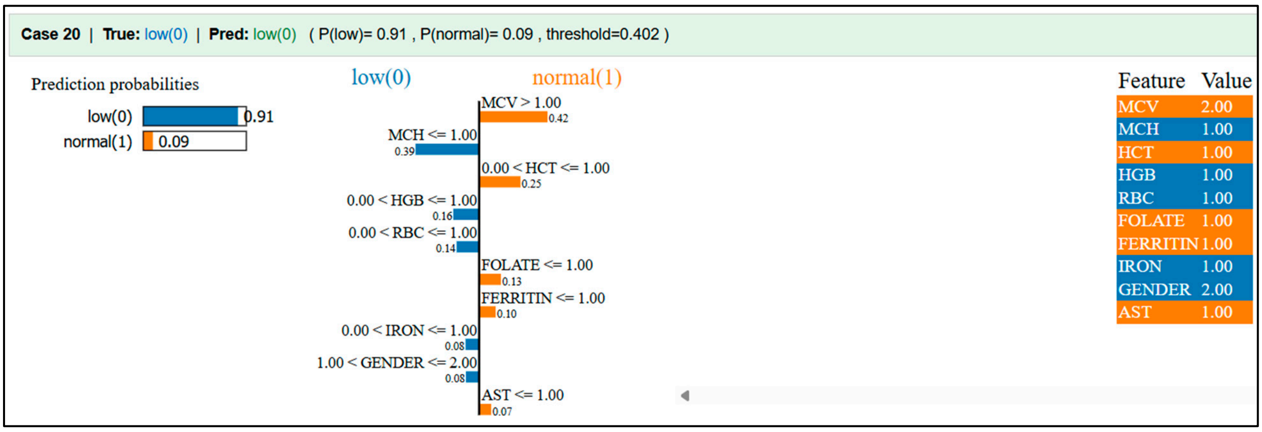

This method generates a local, case-specific interpretation by quantifying how each feature contributes to an individual prediction. Bars plotted in blue represent features that push the prediction toward he “low B12” class (negative direction), whereas bars in orange indicate features that shift the probability to ward the “normal” class (positive direction). The length of each bar reflects the magnitude of its influence on the predicted class probability, and the sign (±) denotes the direction of the effect relative to the model’s decision boundary.
